# Promoting positive maternal, newborn, and child health behaviors through a group-based health education and microfinance program: a prospective matched cohort study in western Kenya

**DOI:** 10.1186/s12884-020-02978-w

**Published:** 2020-05-12

**Authors:** Lauren Y. Maldonado, Julia J. Songok, John W. Snelgrove, Christian B. Ochieng, Sheilah Chelagat, Justus E. Ikemeri, Monica A. Okwanyi, Donald C. Cole, Laura J. Ruhl, Astrid Christoffersen-Deb

**Affiliations:** 1 Academic Model Providing Access to Healthcare (AMPATH), P.O. Box 4606, Eldoret, 30100 Kenya; 2 Department of Medicine, Department of Pediatrics, Massachusetts General Hospital, Boston, MA USA; 3 Moi University School of Medicine, College of Health Sciences, Eldoret, Kenya; 4 Division of Maternal-Fetal Medicine, Department of Obstetrics and Gynaecology, Mount Sinai Hospital, University of Toronto, Toronto, Ontario Canada; 5 LVCT Health, Nairobi, Kenya; 6 Kenya Red Cross, Bomet, Kenya; 7 Dalla Lana School of Public Health, University of Toronto, Toronto, Ontario Canada; 8 Department of Medicine, Indiana University, Indianapolis, IN USA; 9 Department of Obstetrics and Gynaecology, University of British Columbia, Vancouver, British Columbia Canada

**Keywords:** Pregnancy, Community health volunteer, Maternal health, Newborn or infant health, Peer support, Health education, Microfinance, Financial inclusion, Low- and middle-income country (LMIC), Kenya

## Abstract

**Background:**

*Chamas for Change (Chamas)* is a group-based health education and microfinance program for pregnant and postpartum women that aims to address inequities contributing to high rates of maternal and infant mortality in rural western Kenya. In this prospective matched cohort study, we evaluated the association between *Chamas* participation and facility-based delivery. We additionally explored the effect of participation on promoting other positive maternal, newborn and child health (MNCH) behaviors.

**Methods:**

We prospectively compared outcomes between a cohort of *Chamas* participants and controls matched for age, parity, and prenatal care location. Between October–December 2012, government-sponsored community health volunteers (CHV) recruited pregnant women attending their first antenatal care (ANC) visits at rural health facilities in Busia County to participate in *Chamas*. Women enrolled in *Chamas* agreed to attend group-based health education and microfinance sessions for one year; controls received the standard of care. We used descriptive analyses, multivariable logistic regression models, and random effect models to compare outcomes across cohorts 12 months following enrollment, with α set to 0.05.

**Results:**

Compared to controls (*n* = 115), a significantly higher proportion of *Chamas* participants (*n* = 211) delivered in a health facility (84.4% vs. 50.4%, *p* < 0.001), attended at least four ANC visits (64.0% vs. 37.4%, *p* < 0·001), exclusively breastfed to six months (82.0% vs. 47.0%, p < 0·001), and received a CHV home visit within 48 h postpartum (75.8% vs. 38.3%, p < 0·001). In multivariable models, *Chamas* participants were over five times as likely as controls to deliver in a health facility (OR 5.49, 95% CI 3.12–9.64, *p* < 0.001). Though not significant, *Chamas* participants experienced a lower proportion of stillbirths (0.9% vs. 5.2%), miscarriages (5.2% vs. 7.8%), infant deaths (2.8% vs. 3.4%), and maternal deaths (0.9% vs. 1.7%) compared to controls.

**Conclusions:**

*Chamas* participation was associated with increased odds of facility-based delivery compared to the standard of care in rural western Kenya. Larger proportions of program participants also practiced other positive MNCH behaviors. Our findings demonstrate *Chamas’* potential to achieve population-level MNCH benefits; however, a larger study is needed to validate this observed effect.

**Trial registration:**

ClinicalTrials.gov, NCT03188250 (retrospectively registered 31 May 2017).

## Background

Addressing preventable maternal and infant deaths is a significant challenge on the global agenda. As part of Sustainable Development Goal (SDG) 3, the World Health Organization (WHO) and United Nations tasked countries with reducing their maternal mortality ratio (MMR) to less than 70 maternal deaths per 100,000 livebirths and neonatal mortality rate (NMR) to less than 12 deaths per 1000 livebirths by 2030 [1]. This is an ambitious target for Kenya, where the MMR and NMR are 362 per 100,000 and 22 per 1000 livebirths, respectively [2]. Evidence-based strategies that enhance the practice of lifesaving Maternal, Newborn and Child Health (MNCH) behaviors are urgently needed to meet these SDG targets [3, 4].

Per WHO and Republic of Kenya Ministry of Health (MOH) recommendations, these health behaviors may include: delivering in health facilities with skilled birth attendants (SBA), attending at least four focused antenatal care (ANC) visits, receiving a community health volunteer (CHV) home visit within 48 h of delivery, and exclusively breastfeeding (EBF) infants to six months [5–11]. Promoting access to and increasing use of long-term family planning (FP) methods may decrease risk for maternal and perinatal morbidity and mortality by allowing women to limit and space pregnancies [12–14]. Further, ensuring infants receive the Oral Polio Vaccination at birth (OPV0) may increase protection against infectious disease mortality during the first year of life [15].

In rural Kenya, only half of women deliver in a health facility with an SBA (47%), attend at least four ANC visits (51.3%), and receive a CHV visit within the first 48 h after delivery (53%) [2]. EBF beyond the initial months postpartum is uncommon, lasting a median of 3.4 months among rural populations [16]. Less than half of all women currently use a modern FP method (39.1%), and among users, less than 10% select a long-term or permanent method [2]. From an equity perspective, poor MNCH outcomes are disproportionate across socioeconomic strata. Access to care is generally correlated with economic accessibility and women of lower socioeconomic status often encounter greater barriers to accessing high quality care [2, 17].

In addition to promoting positive MNCH behaviors, one of the key enablers in meeting the SDGs is financial inclusion. The World Bank defines financial inclusion for individuals as “access to useful and affordable financial products and services that meet their needs – transactions, payments, savings, credit and insurance – delivered in a responsible and sustainable way.” [18] The ability to store money, transfer payments and access loans is increasingly recognized as a vital strategy to overcome financial barriers to health. In Kenya, however, it is estimated that up to one-third of the population is excluded from the formal financial sector [19]. This is particularly true among women in rural Kenya, who are disproportionately excluded from participating in formal income generating activities, making it difficult to adequately finance health-related expenditures [19]. This continued pattern of exclusion of poor and rural women perpetuates their precarious financial and social position.

To address inequities contributing to high rates of maternal and infant mortality in rural western Kenya, the Academic Model Providing Access to Healthcare (AMPATH), in partnership with the Government of Kenya (GOK), launched *Chamas for Change (Chamas)* in 2012. This CHV-facilitated program offers pregnant women free health and microfinance education in a supportive group setting during the antenatal and postpartum period. Translated from Kiswahili as ‘groups with purpose,’ “chamas” have a longstanding presence in East Africa [20]. These groups are highly gendered institutions that women have relied on for centuries for social support and resource pooling [21]. Using this cultural script, our solution combines best practices from women’s health and microfinance programs to create an integrated model that strives to not only improve health outcomes, but also interrupt cycles of poverty by empowering women to live financially-secure lives.

In this article, we report findings from a prospective matched cohort study in Busia County, Kenya. We evaluated the association between *Chamas* participation and facility-based delivery. We additionally explored the effect of program participation on promoting other MNCH behaviors, namely: attending at least four ANC visits, receiving a CHV home visit within 48 h postpartum, EBF to six months, using a modern FP method, using a long-term FP method, and vaccinating infants with OPV0 at birth. We hypothesized participating in *Chamas* would increase the odds of facility-based delivery and the practice of other positive MNCH behaviors compared to receiving the standard of care.

## Methods

### Study setting and design

We conducted our study in Bunyala, a rural sub-county in Busia, Western Province, Kenya. We selected Bunyala for two primary reasons: (1) the MMR and NMR are much higher than national estimates, and (2) the MOH demonstrated strong interest and support of AMPATH’s programs and collaborations. Women and infants in Western Province suffer from the second highest maternal and neonatal mortality rates nationally [2, 22, 23]. In Busia County, the most recent estimate for infant mortality rate (IMR) is exceedingly high at 125.9 per 1000 live births [24]. In Bunyala, MNCH activities, including antenatal and postpartum care led by the GOK and supported by AMPATH, exist across 16 community units and 8 MOH health facilities.

To evaluate our primary and secondary outcomes of interest, we used a prospective, matched-cohort study design. We compared outcomes between a cohort of *Chamas* participants recruited during their first ANC visits at public health facilities in Bunyala and controls receiving the standard of care identified through health facility registers, matched for age, parity, and prenatal care location. We followed both cohorts prospectively for one year and recorded outcome data 12 months following enrollment for all participants.

### Participant selection

**Fig. 1:**
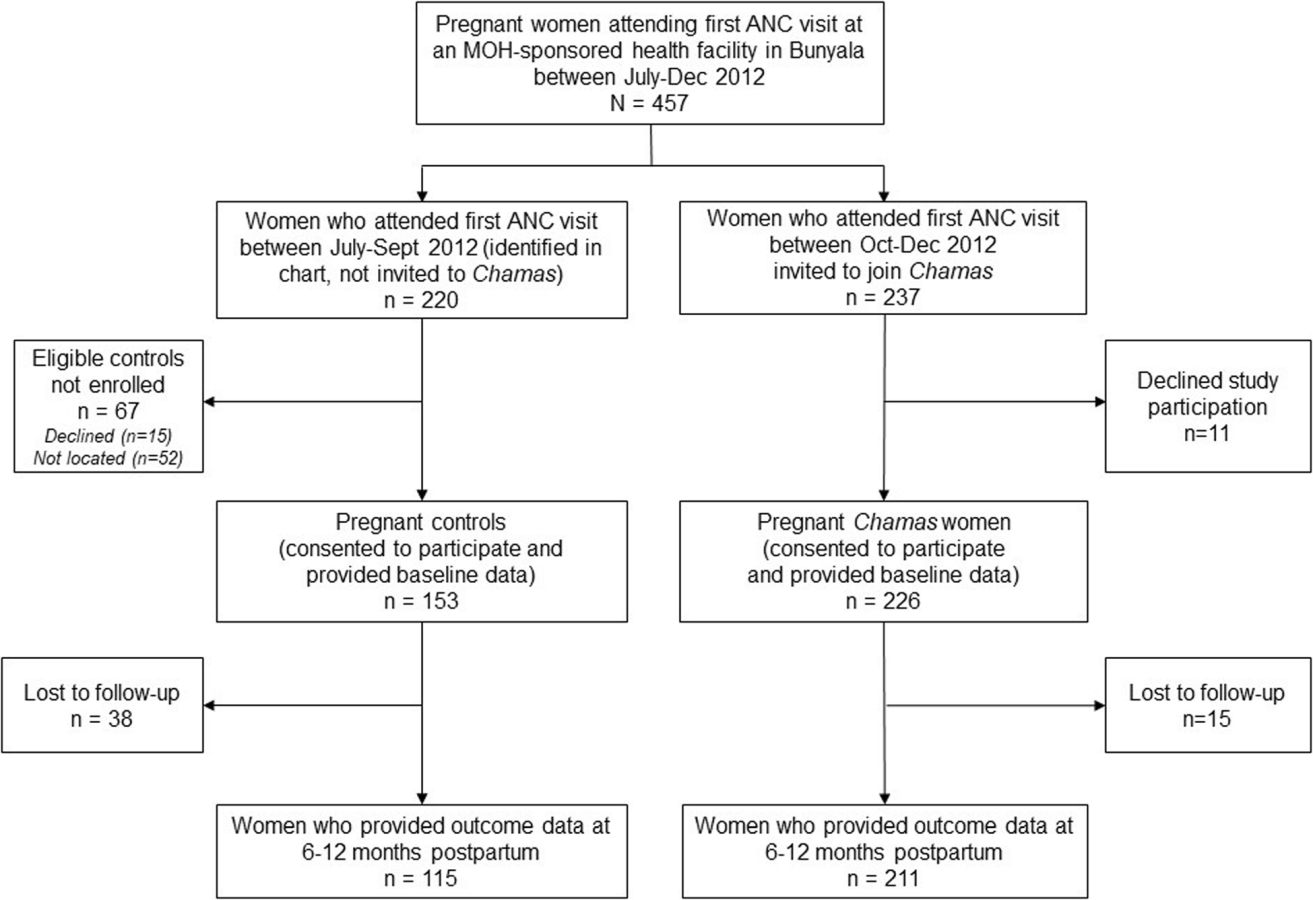
Study flow diagram

We used a facility-based recruitment strategy to enroll women to our intervention cohort. We invited all pregnant women attending their first ANC visit at an MOH-sponsored health facility in Bunyala between October–December 2012 to enroll in the *Chamas* program and to participate in this study (Fig. 1). We did not exclude women based on any sociodemographic or reproductive health factors including age, education-level, employment-status, marital status, parity, or prior history of facility delivery.

To recruit our control cohort, we retrospectively identified pregnant women who attended the same health facilities for their first ANC visits in the three months preceding *Chamas* enrollment (July–September 2012) from clinic registers. We matched controls based on three criteria: age, parity, and prenatal care location (health facility). We tasked CHVs with approaching eligible women at their homes, if they provided an address; we then enrolled women who were successfully located and agreed to participate (Fig. 1). Women in both cohorts provided baseline sociodemographic and reproductive health data at the time of enrollment and consented to complete a 12 month follow-up survey (6–12 months postpartum).

### Community health volunteers in Kenya

*Chamas* leverages CHVs to deliver health and microfinance education in a safe and familiar setting. As delineated by Kenya’s community health strategy, CHVs are members of the community, nominated from within, who are tasked with improving the community’s health and well-being as well as linking individuals to primary health care services [25]. CHVs are considered part-time government volunteers and are supervised by Community Health Extension Workers (CHEWs), salaried frontline healthcare providers integrated within government health facilities [26]. CHV facilitators across both study arms were connected to eight health facilities, specifically: five dispensaries, two health centers, and one sub-county level hospital.

Nationally, the Kenyan government delineates a CHV’s scope of work to include: monthly household visits within a defined catchment area of 20 households in rural areas and 100 households in urban areas [27]. During routine visits, CHVs collect basic health information, identify health problems, and refer individuals needing additional services to health facilities. All CHVs are required to complete a 10-day, MOH-led basic training session prior to beginning work during which they are introduced to a broad array of health topics, including MNCH. With regard to MNCH, CHVs are provided with a handbook that covers basic information on caring for mothers during and after pregnancy, instructions on facilitating the creation of an individualized birth plan, and lists of specific health behaviors they are encouraged to promote (i.e. attend ANC, deliver in health facilities, adopt family planning) [27]. They are also expected to recognize danger signs during pregnancy as well as perform basic nutritional assessments, aid in growth monitoring, and recognize when infants require further evaluation for malnutrition. This basic training is often supplemented by technical training that aligns with local priorities; however, technical sessions are variable and often implemented by local governments or non-governmental organizations [27].

In September 2012, we selected 32 GOK sponsored CHVs to participate in an additional four-day technical training session on *Chamas*, sponsored by AMPATH and the Busia County MOH. During these sessions, we trained attendees on how to deliver our evidence-based health curriculum using an illustrated flipchart, facilitate participatory group discussions, and equip program participants with basic microfinance literacy and skills. In addition to conducting didactic sessions, CHVs also received additional training on basic health interventions, such as taking vitals, assessing for hemorrhage and signs of infection at the 48-h postpartum home-visit, supporting mothers in exclusively breastfeeding, counselling participants on options for family planning, and adopting safe sleep practices. Throughout the year, CHVs attended regularly scheduled check-in meetings (at months 1–4, 6, 9, and 12) with AMPATH implementation leads to provide feedback, as well as receive additional mentorship and support.

### Intervention description

Women attending *Chamas* convened twice per month for 12 months to attend a total of 24 CHV-facilitated group health education and microfinance sessions. Each group was typically comprised of 15–30 women and each session consisted of a 60 to 90-min participatory lesson on one health (i.e. antenatal care, family planning) and one social (i.e. intimate partner violence, microfinance literacy) topic (Table 1). CHVs used an illustrated flip-chart with an accompanying discussion guide to facilitate sessions. Upon joining the program, women agreed to practice key MNCH behaviors, namely to: deliver in a health facility, attend at least four ANC visits, EBF to six months, receive a CHV home visit within 48 h of delivery, consider a long-term method of FP, ensure their infant received OPV0, and save money to finance health expenditures. Each group also delineated personal goals they wished to accomplish during the program.

**Table 1:** Health and Social Topics for *Chamas for Change* First-Year Curriculum^a^

Lesson	Health Topic	Social Topic
**1**	Importance of antenatal and postnatal care	Goals of the *Chamas for Change* program
**2**	Physical exercise during pregnancy	Table banking (Saving and Loans)
**3**	Anemia during pregnancy	National Hospital Insurance Fund (NHIF)
**4**	Danger signs during pregnancy and after delivery	Nutrition during pregnancy
**5**	Importance of facility delivery	Involving male partners during pregnancy and while raising children
**6**	Preventing maternal-child transmission of infections	Supporting the birth of a child in your Chama
**7**	Negative pregnancy outcomes (losing an infant)	Post-delivery welfare (up to 12 months of age)
**8**	Complications during pregnancy and delivery (i.e. obstructed labor)	Creating a budget
**9**	Postpartum depression	Setting routines for the infant: sleeping and eating
**10**	Newborn danger signs (4 h to 2 weeks)	Promoting a good relationship with your Mother-in-law and Sister-in-law
**11**	Exclusively breastfeeding for 6 months	Home hygiene
**12**	Infant growth monitoring and under-5 immunizations	Disclosing HIV status to your family
**13**	Kangaroo Care	Reducing stigma towards members in the community with HIV
**14**	Back to sleep/co-sleeping	Cooking in clean air
**15**	Family Planning: Coil/Uterine Copper Device	Farming and rearing livestock
**16**	Family Planning: Jadelle, Implanon, Nexplanon	Clean water
**17**	Family Planning: Male and Female Condoms	Intimate Partner Violence (IPV)
**18**	Infant growth and development	Adolescent pregnancies
**19**	Complementary feeding for infants	Importance of female education
**20**	Basic first aid: choking and burns	Promoting a good relationship with your husband in the home
**21**	Pediatric diseases under surveillance: Measles, Polio, Pneumonia, and Scabies	Mutual sexual satisfaction between a man and a woman
**22**	Diarrheal Diseases	Preparing to take your child to school (preparing for pre-school)
**23**	Cervical Cancer Screening: Overcoming fears and misconceptions	Group conflict resolution
**24**	Malaria	Children with developmental delays

^a^The Year 1 Chamas curriculum is comprised of 24 lessons (one health and one social topic) delivered over the span of 12 months. Lessons are facilitated by community health volunteers in a group-based setting using illustrated flip-charts and discussion guides

Following lessons, members elected to participate in a table-banking program called “Group Integrated Savings for Health and Empowerment” (GISHE). GISHE is an adaptation of the Catholic Relief Services’ *Savings and Internal Lending* model, which encourages a savings-led, group-based microfinance scheme [28]. We deemed participation optional to avoid excluding women that could not afford to contribute the minimum 50 KSH (0.50 USD) share per meeting. Members contributed up to ten times the amount of the minimal share at each *Chamas* session. The group provided loans that amounted to a multiple of the individual member’s savings and returned a dividend payment based on interest accrued at the end of the year. Profits generated were distributed to the entire group in amounts proportional to individual shares contributed.

We designed *Chamas* in collaboration with the GOK and county-level MOH representatives to ensure support and investment from local community members. The *Chamas* curriculum was designed by a diverse group of stakeholders including AMPATH researchers, community members, and local MOH representatives. The curriculum was designed with the intent to highlight evidence-based recommendations by international authorities (i.e. WHO), bolster training provided through the current CHV handbook, and respond directly to the needs of and questions asked by the local community. We sought feedback throughout curriculum development through conducting focus group discussions with community representatives. This pilot study served as a debut for this curriculum.

Our control cohort received the current standard of care as delineated by the MOH (described under *Community Health Volunteers in Kenya*). In contrast to *Chamas* participants, they received monthly, individual CHV household visits, but did not participate in structured, evidence-based health education and microfinance sessions nor experience the group-based format offered by the program.

### Data collection and study variables

We collected baseline and outcome data at two time-points for all participants using paper-based, structured, data collection forms (Additional File 1). We tasked AMPATH research assistants trained in data entry with collecting data at both time-points. We recorded baseline data on sociodemographic and reproductive health information at enrollment and collected outcome data at 12 months follow-up (6–12 months postpartum). Interview location depended on the study time-point and cohort assignment. We collected baseline data on intervention participants at health facilities and on controls at participant homes on the day of enrollment. We collected all outcome data at participant homes. During both time-points, we made every effort to collect data individually and privately so as to minimize potential for response bias.

Our primary outcome was the odds of facility-based delivery. Our secondary MNCH outcomes included: the relative proportion of women who attended at least four ANC visits, received a CHV home-visit within 48 h postpartum, EBF to 6 months, adopted a modern FP method, and adopted a long-term or permanent FP method. We additionally assessed the relative proportion of infants that received OPV0 at birth across cohorts. Where possible, we extracted data from Maternal and Child Health (MCH) booklets. If women did not have their MCH booklet available or if booklets missed data, we asked participants to self-report answers.

To assess the modifying effect of covariates we collected sociodemographic and reproductive health information, including: age, education level, employment status, marital status, parity, prior facility delivery (among those who previously delivered), and facility location of first ANC visit. Maternal age may worsen maternal and fetal outcomes, increasing the propensity of older women to seek care or establish contact with health facilities earlier in pregnancy [29]. Sociodemographic characteristics such as education level, employment status and marital status may impact the likelihood of facility delivery as these variables serve as proxies for socio-economic status. We defined “employment” as earning the national daily minimum wage of 450 Kenyan Shillings and allowed participants to select a categorical descriptor (i.e. housewife/unemployed, self-employed, agricultural worker, other) [26]. Previous studies demonstrate women of lower socio-economic status or lower levels of education are less likely to deliver in facilities [30]. Further, reproductive health characteristics such as parity and prior facility delivery may positively or negatively impact a woman’s likelihood of returning to facilities, based on experiences with the health system [31, 32]. Lastly, we collected first ANC visit facility location to address potential area-level variance on the likelihood of facility delivery.

Though not powered to detect significant differences, we assessed pregnancy-related morbidity and mortality outcomes as well as microfinance data using program monitoring logs recorded by CHVs. These outcomes specifically included: the gestational age (GA) at delivery, the incidence of miscarriage (defined as loss of fetus less than 28 weeks gestation) and stillbirth (defined as loss of fetus between 28 weeks and delivery), as well as the incidence of infant and maternal mortality. We restricted microfinance outcomes to the *Chamas* cohort and these included: the proportion participating in GISHE, individual loans received, group savings accumulated, and general categories of investment (i.e. school-fees, health expenditures, small businesses). CHVs reported these data monthly to trained research assistants, who electronically transcribed and uploaded outcomes to an encrypted database.

### Sample size determination

To calculate our estimated sample size, we assumed 55% of women who attended at least one ANC visit delivered in a health facility and an intra-class correlation coefficient of 0.34, which accounts for population-level variance due to area-level effects (i.e. contact and proximity to the health system and CHVs, clustering by health facility catchment area) [2, 33]. With these assumptions, we determined a 2:1 sample of 240 (156 *Chamas* and 84 Control) participants adequate to detect a 20% difference in the proportion of facility deliveries between intervention and control groups, with a type I error rate (α) of 0.05 and power of 85%. We assumed a 10% loss to follow-up and established a final target sample size of 267 total participants.

### Data analysis

We tabulated frequencies and calculated descriptive statistics comparing socio-demographic and reproductive health variables between *Chamas* participants and controls. For all bivariate analyses, we used student’s T tests for continuous variables, Mann-Whitney U tests for continuous variables with non-normal distributions, two-sample Z-score tests for proportions, and Chi-square tests for categorical variables.

Multivariable nested models were used to test the association between *Chamas* participation and facility delivery independently, with successive inclusion of covariates, namely: age, education level, employment status, marital status, parity, and prior facility delivery. We examined age as a continuous variable. We collapsed education level into a three-level categorical variable (none-some primary, completed primary, some-completed secondary), and employment (unemployed vs. employed), marital status (single/separated/divorced vs. married), parity (nulliparous vs. multiparous), and prior facility delivery into dichotomous variables. We performed complete case analyses and excluded records with missing data on the primary outcome variable or covariates.

Random effects models, employing the same nested-inclusion technique described above, tested for significant area-level variance as determined by prenatal care location. We additionally ran an interaction model with ANC attendance (dichotomous variable, < 4 visits vs. ≥4 visits) and *Chamas* participation as we hypothesized mothers attending at least four ANC visits were more likely to deliver in a health facility than those who attended fewer than four visits [32]. We decided a priori to conduct an additional sensitivity analysis restricting our intervention sample solely to *Chamas* women who participated in GISHE to examine the impact of combined effect of health education and microfinance participation on MNCH intervention uptake. We conducted all statistical analyses using Stata version 13.1 (StataCorp, College Station, Texas) with α set to 0.05.

### Ethical consideration and trial registration

Our study received ethics approval from the Institutional Research Ethics Committee at Moi Teaching and Referral Hospital (IREC/2013/76), the Office of Research Administration at Indiana University (#1306011628), and the Research Ethics Board at the University of Toronto (# 2907). We obtained written informed consent from all participants prior to data collection. We retrospectively registered this study with ClinicalTrials.gov (NCT03188250). No substantial changes were made to the study design or outcomes following participant enrollment.

## Results

Between October–December 2012, we identified and invited 237 women attending their first ANC visits to join *Chamas*; we simultaneously identified and attempted to contact 220 women who attended their first ANC visits in the three months preceding enrollment using clinic registers (Fig. 1). Most eligible women who were successfully contacted across both the control (153/168, 91.2%) and intervention (226/237, 95.4%) arms agreed to participate in this study. Loss to follow-up rates across the control and intervention arms at end-line assessment were 24.8 and 6.6%, respectively. Results are solely reported for our final sample size of 326 women (*n* = 115 control, *n* = 211 intervention participants) who contributed outcome data 12 months following enrollment (between 6 and 12 months postpartum).

### Participant demographics

Baseline sociodemographic and reproductive health characteristics by participant group are presented in Table 2. Overall, our cohort and intervention groups were well-matched with few significant differences. Participants averaged 25.2 years of age. Most completed primary school, were married, and attended their first ANC visit at 22.1 weeks gestation. A significantly higher proportion of women in the control cohort were unemployed (56.5% vs. 40.3%, *p* < 0.05) and previously delivered a live-born infant (100% vs. 87.7%, p < 0.05) than women in *Chamas*. Among women with a previous delivery, a significantly higher proportion of *Chamas* participants delivered in a health facility (65.3% vs. 47.8%, *p* < 0.05); however, we reported missing data for nearly 20% of controls for this covariate.

**Table 2:** Baseline sociodemographic and reproductive health characteristics by participant group for study population (*n* = 326)

Variable	Study Population (n = 326)	*Chamas* (n = 211)	Control (n = 115)
	M ± SD or % (n)	M ± SD or % (n)	M ± SD or % (n)
**Age**	25.2 ± 4.8	25.2 ± 5.0	25.1 ± 4.5
**Education level**			
None-some primary	14.7 (48)	11.8 (25)	20.0 (23)
Completed Primary	73.6 (240)	73.9 (156)	73.0 (84)
Some secondary	6.1 (20)	8.1 (17)	2.6 (3)
Completed secondary	5.6 (18)	6.2 (13)	4.4 (5)
Employment^*^			
Housewife (unemployed)	46.0 (150)	40.3 (85)	56.5 (65)
Self-employed	30.3 (99)	36.0 (76)	20.0 (23)
Agricultural worker	16.3 (53)	18.0 (38)	13.0 (15)
Other	7.4 (24)	5.7 (12)	10.5 (12)
**Marital Status**			
Married	86.8 (283)	85.3 (180)	89.6 (103)
Single/Separated/Divorced	13.2 (43)	14.7 (31)	10.4 (12)
Parity^*^			
Mean (SD)	2.8 ± 1.8	2.5 ± 1.8	3.0 ± 1.7
Parous	92.0 (300)	87.7 (185)	100.0 (115)
Nulliparous	8.0 (26)	12.3 (26)	0.0 (0)
Prior facility delivery^*,a,b^			
Yes	58.7 (176)	65.3 (121)	47.8 (55)
No	27.7 (83)	25.0 (46)	32.2 (37)
***Gestational age at first ANC visit (weeks)***	22.1 ± 8.5	22.4 ± 8.9	21.8 ± 8.0
**First ANC visit location**^b^			
Port Victoria	27.6 (90)	25.6 (54)	31.3 (36)
Budalangi	8.3 (27)	7.6 (16)	9.6 (11)
Sirimba	11.3 (37)	12.8 (27)	8.7 (10)
Sisenya	12.9 (42)	12.8 (27)	13.0 (15)
Mukhobola	13.2 (43)	11.9 (25)	15.7 (18)
Rukala	15.6 (51)	16.1 (34)	14.8 (17)
Bulwani	4.9 (16)	5.2 (11)	4.4 (5)
Osieko	3.1 (10)	3.3 (7)	2.6 (3)
Other	2.1 (7)	3.3 (7)	0.0 (0)

^*^Significant *p* < 0.05

^a^Among those with previous delivery (*Chamas* group = 185; control group = 115)

^b^Missing data: prior facility delivery *n* = 18 (*Chamas*), *n* = 23 (Control); first ANC location n = 3 (*Chamas*)

### Practice of positive MNCH behaviors

Results for the practice of positive MNCH behaviors by cohort are presented in Table 3. Compared to controls, a significantly higher proportion of *Chamas* participants delivered in a health facility (84.4% vs. 50.4%, *p* < 0.05), attended at least 4 ANC visits (64.0% vs. 37.4%, p < 0.05), received a CHV home visit within 48 h postpartum (75.8% vs. 38.3%, p < 0.05), and exclusively breastfed to 6 months postpartum (82.0% vs. 47.0%, p < 0.05). Though not statistically significant, a higher proportion of *Chamas* participants adopted a modern method of contraception (58.2 vs. 55.6%, *p* = 0.46) and among method adopters, a higher proportion chose a long-acting or permanent FP method (66.7 vs. 62.5%, *p* = 0.58) as compared to controls. Lastly, a higher proportion of infants born to women participating in *Chamas* received the OPV0 immunization at birth (91.9% vs. 85.2%, *p* = 0.41). Missing values comprised less than 10% of each cohort across all outcomes measured.

**Table 3:** Practice of maternal, newborn and child health behaviors by participant group for study population (n = 326)

Health Behavior	Study Population (n = 326)	Chamas (n = 211)	Control (n = 115)
	M ± SD or % (n)	M ± SD or % (n)	M ± SD or % (n)
Delivered in a facility with skilled birth attendant^*,b^			
Yes	72.4 (236)	84.4 (178)	50.4 (58)
No	22.7 (74)	12.8 (27)	40.9 (47)
Attended ≥ 4 ANC visits^*,b^			
Yes	54.6 (178)	64.0 (135)	37.4 (43)
No	43.9 (143)	33.7 (71)	62.6 (72)
Received CHV 48-h postpartum home visit^*,b^			
Yes	62.6 (204)	75.8 (160)	38.3 (44)
No	32.5 (106)	19.9 (42)	55.7 (64)
Exclusively breastfed ≥ 6 months^*,b^			
Yes	69.6 (227)	82.0 (173)	47.0 (54)
No	22.7 (74)	11.9 (25)	42.6 (49)
**Adopted*any*modern family planning method or permanent method (oral contraceptives, injections, IUD, implant, tubal ligation)**^b^			
Yes	57.4 (187)	58.2 (123)	55.6 (64)
No	41.7 (136)	40.7 (86)	44.3 (51)
**Adopted a*long-term or permanent method*of family planning (IUD, implant, tubal ligation)**^**a**^			
Yes	65.0 (122)	66.7 (82)	62.5 (40)
No	34.8 (65)	33.3 (41)	47.1 (24)
**Infant received OPV0 immunization**^b^			
Yes	89.5 (292)	91.9 (194)	85.2 (98)
No	3.4 (11)	2.8 (6)	4.4 (5)

^*^Significant *p* < 0.001

^a^Among women who answered “yes” to adopting any modern family planning method (*n* = 187)

^b^Missing data: facility delivery *n* = 6 (*Chamas*), *n* = 10 (Control); ANC visit attendance *n* = 5 (*Chamas*); 48-h CHV home visit *n* = 9 (*Chamas*), *n* = 7 (Control); Exclusively breastfed to 6 months *n* = 13 (*Chamas*), n = 12 (Control); Any family planning n = 2 (*Chamas*); OPV0 immunization n = 11 (*Chamas*), *n* = 12 (Control)

### Maternal and infant morbidity and mortality

Women in *Chamas* delivered at a significantly older gestational age than controls (39.4 ± 2.7 vs. 35.5 ± 8.9, *p* < 0.001). Of note, 33% (*n* = 38) of the control cohort missed data for this outcome, limiting interpretability of this result. Further, women in *Chamas* experienced a lower proportion of stillbirths (0.9% vs. 5.2%), miscarriages (5.2% vs. 7.8%), infant deaths (2.8% vs. 3.4%), and maternal deaths (0.9% vs. 1.7%) as compared to controls.

### Association between Chamas participation and health facility delivery

Fully adjusted results from our multivariable analyses are presented in Table 4. We excluded 19 participants (5.8% of sample) from our analyses as they missed primary outcome data on facility delivery or on a covariate. Excluded participants did not substantially differ in sociodemographic or reproductive health characteristics from those included in our analysis. In our unadjusted model, *Chamas* participation was associated with over five times the odds of delivering at a health facility compared to controls (OR 5.49, 95% CI 3.12–9.64, *p* < 0.001). This effect was only slightly attenuated after controlling for age, education level, employment, marital status, parity, and prior facility delivery (OR 5.07, 95% CI 2.74–9.36, p < 0.001). Following adjustment, prior facility delivery was the only significantly associated covariate; those with a prior facility delivery were roughly four times as likely as those without to deliver in a health facility (OR 4.31, 95% CI 2.25–8.25, p < 0.001).

**Table 4:** Multivariable logistic regression model of association between *Chamas* participation and facility delivery adjusted for sociodemographic and reproductive health covariates (*n* = 307)^a^

Variable	Facility delivery with skilled birth attendant		
	OR	(95% CI)	*p*-value
***Chamas*participation**			
Did not participate in *Chamas*	–	–	–
Participated (unadjusted)	5.49	(3.12, 9.64)	< 0.001
Participated (adjusted)	5.07	(2.74, 9.36)	< 0.001
**Age (years)**	1.00	(0.93, 1.08)	0.92
**Education level**			
None-some primary	–	–	–
Completed primary	1.22	(0.56, 2.66)	0.63
Some-completed secondary	3.24	(0.74, 14.17)	0.12
**Employment**			
Housewife (unemployed)	–	–	–
Self-employed/Agricultural Worker/Other	1.38	(0.74, 2.55)	0.31
**Marital Status**			
Single/Separated/Divorced	–	–	–
Married	1.56	(0.52, 4.63)	0.43
**Parity**			
Primiparous	–	–	–
Multiparous	1.10	(0.18, 6.89)	0.92
**Prior facility delivery**			
No	–	–	–
Yes	4.31	(2.25, 8.25)	< 0.001

^a^Complete cases only; *n* = 19 participants missing data on primary outcome or covariate

We used random effects modelling to determine whether significant area-level variance due to prenatal care location impacted the likelihood of facility delivery (Table 5). We grouped women according to the health facility they attended for their first antenatal visit; among our cohort of women, they sought care at eight different health facilities (five dispensaries, two health centers, and one sub-county level hospital) (Table 2). Our null model revealed a significant amount of area-level variance (σ_u_^2^ = 0.30 ± 0.24, *p* < 0.05) in the odds of facility delivery. Following adjustment for covariates, the variance remained statistically significant (σ_u_^2^ = 0.44 ± 0.39, p < 0.05); however, the association between *Chamas* participation and adjusted odds of delivering in a health facility was materially unchanged (OR 5.60, 95% CI 2.91–10.80, *p* < 0.001).

**Table 5:** Nested random effects models of association between *Chamas* participation and facility delivery controlling for prenatal care location (n = 307)^a^

Model variance estimates	*Random Effects Model*		
	Null	Unadjusted	Adjusted
σ_u_^2^ (SE)	0.30 (0.24)	0.51 (0.40)	0.44 (0.39)
ρ (SE)	0.08 (0.06)	0.14 (0.09)	0.12 (0.09)
p-value^□^	0.01	< 0.01	0.01
−2 log likelihood	330.23	291.10	267.40
**Covariates**	**OR (95% CI)**	**OR (95% CI)**	**OR (95% CI)**
***Chamas*participation**			
Did not participate in *Chamas*	N/A	–	–
Participated	N/A	6.40 (3.44, 11.76)^ỻ^	5.60 (2.91, 10.80) ^††^
**Age (years)**	N/A	N/A	1.00 (0.92, 1.08)
**Education level**			
None-some primary	N/A	N/A	–
Completed primary	N/A	N/A	1.22 (0.54, 2.74)
Some-completed secondary	N/A	N/A	3.28 (0.73, 14.75)
**Employment**			
Housewife (unemployed)	N/A	N/A	–
Self-employed/Agricultural Worker/Other	N/A	N/A	1.47 (0.76, 2.85)
**Marital Status**			
Single/Separated/Divorced	N/A	N/A	–
Married	N/A	N/A	1.35 (0.44, 4.15)
**Parity**			
Primiparous	N/A	N/A	–
Multiparous	N/A	N/A	1.03 (0.15, 6.95)
**Prior facility delivery**^**□**^			
No	N/A	N/A	–
Yes	N/A	N/A	4.16 (2.09, 8.27) ^††^

^a^Complete cases only; n = 19 participants missing data on primary outcome or covariate

^□^Likelihood ratio test, ρ = 0

^††^Significant *p* < 0.001

Finally, we tested for interaction between ANC attendance and *Chamas* participation with a likelihood ratio test and did not find an interaction effect based on an a priori significance level of 0.05 (analyses not shown).

### Effect of microfinance participation

Among all women participating in *Chamas*, 71.8% (*n* = 152) also participated in GISHE. A significantly higher proportion of women participating in GISHE completed at least some secondary school (18.9% vs. 3.4%, *p* < 0.05) and were employed (63.5% vs. 48.3%, p < 0.05) than those who chose not to participate. On average, six group members received loans per meeting, varying from 200 to 2000 KSH (2–22 USD). Women primarily used loans to pay for school fees, business start-up costs and health service-related fees. All 16 *Chamas* groups generated adequate funds to repay group start-up costs of 5000 KSH (50 USD). There were no statistically significant differences in either the primary (facility delivery) or secondary outcomes when we compared GISHE participants to those not participating within the *Chamas* cohort.

We conducted an additional sensitivity analysis restricting our intervention sample solely to *Chamas* women who participated in GISHE (*n* = 152) to examine the impact of both health education and microfinance participation on MNCH intervention uptake. Results generated by this model were materially unchanged from those generated by our multivariable models (analyses not shown).

## Discussion

### Major findings

In this study, we evaluated the association between *Chamas* participation and facility-based delivery in rural western Kenya. We additionally explored the effect of program participation on promoting other positive MNCH behaviors. We affirmed our hypothesis by demonstrating *Chamas* participants had a significantly higher odds of achieving a facility-based delivery as compared to women receiving the standard of care. Additionally, larger proportions of *Chamas* participants attended at least four ANC visits, breastfed exclusively to six months, and received a 48-h postpartum CHV home-visit. Though not statistically significant, a larger proportion of *Chamas* participants adopted a long-term or permanent FP method and immunized infants with OPV0 compared to controls. Taken together, these results suggest *Chamas* may offer a promising strategy to promote positive MNCH behaviors needed to achieve SDG targets by 2030.

Our random effect models revealed significant area-level variance based on location of prenatal care and likelihood of facility delivery. Of interest, the variance remained statistically significant after controlling for covariates. This finding suggests there may be unobserved compositional effects within *Chamas* groups *-* or contextual effects between them - that explain some of the remaining variance in our primary outcome. The specific characteristics of our program that promote health care access are out of scope for the present study; however, future work may elucidate the causal pathways through which *Chamas* involvement influences uptake of MNCH services.

Further, we examined the combined effect of health education and microfinance participation on achieving MNCH outcomes. Recent literature suggests integrating microfinance schemes within women’s health education or service delivery programs may enhance health outcomes [34, 35]. Though our sensitivity analyses revealed no significant difference in results based on microfinance participation, non-GISHE participants comprised less than half of the intervention cohort. Additionally, we did not assess participation in microfinance activities apart from GISHE across cohorts though crude estimates suggest a substantial portion of the population (nearly 30% in some rural western Kenyan counties) is involved in table-banking [36, 37]. It is possible participation in other microfinance schemes may nullify the effect of GISHE participation; however, additional research is needed to clarify this association. We also plan to clarify use of GISHE funds and to investigate the impact of investing in health-related expenditures.

### Strengths and limitations

Our study has several notable strengths. We used a prospective, matched-cohort design to ensure reasonable comparisons between *Chamas* participants and controls. Our cohorts were relatively well-matched, with adequate sample sizes to detect significant differences in our primary and secondary outcomes. We designed the *Chamas* program in collaboration with GOK and county-level MOH representatives, which offers confidence in long-term support and investment from local stakeholders. Further, our study aligns with current task-sharing recommendations by providing evidence to support the mobilization of CHVs to address unmet needs of pregnant and postpartum women [38]. *Chamas* also builds upon preceding group-based programs to enhance MNCH in resource-limited settings. In resource-limited settings, CHV-based efforts to promote health education through women’s groups have demonstrated substantial promise in improving MNCH outcomes [39–44]. *Chamas* integrates these strategies to provide tailored programming that addresses needs of rural Kenyan women and infants. Collectively, these findings underscore a need for additional work to clarify whether there may be a synergistic effect in combining MNCH education and microfinance participation on population-level health and financial outcomes [39–44]. Moreover, regional and national efforts to address MNCH are increasing in Kenya as evidenced by publicly-funded insurance initiatives such as *Linda Mama* and the *National Hospital Insurance Fund* [45]. These services strive to ensure pregnant women and infants have access to quality and affordable health services by promoting universal health coverage. To clarify associations between *Chamas* participation and concurrent initiatives aimed at improving MNCH outcomes, we plan to assess and control for additional demographics such as health insurance enrollment in future studies.

There are several noteworthy limitations of our study. First, though we were able to detect significant differences in our primary health-related outcomes of interest, we did not adequately power our study to determine the effect of *Chamas* participation on maternal and infant morbidity and mortality. We plan to examine these outcomes more thoughtfully in future studies with more reliable measures to assess morbidity (i.e. infant birthweight). Second, though we intended to conduct end-line assessments with all study participants around 6–12 months postpartum, we assessed most participants closer to 9–15 months postpartum due to logistical constraints. This time-lag may have increased risk for recall bias, particularly when objective data were not available from MCH Booklets. Third, our recruitment model introduced significant challenges that likely limited our control cohort size and introduced selection bias. By recruiting women from ANC facilities, we may have inadvertently excluded the most vulnerable women in the community who experience barriers to accessing care. Future strategies that combine both facility and community-based recruitment methods should be explored. Fourth, we experienced retention-related challenges as many families moved or did not supply reliable addresses; as such, we experienced high lost to follow-up rates particularly among our control cohort. Instituting a more reliable tracking method will be essential to increase cohort size and limit loss-to-follow-up in future studies. Fifth, though we sought to decrease the risk of response bias by collecting participant data privately, we speculate some women over-reported positive health behavior adherence due to pressure associated with social desirability. Sixth, our paper-based assessments resulted in substantial missing data on both demographic and outcome indicators. As such, we were unable to include all participants in our analysis of our primary outcome. We intend to use digital-based data collection methods to improve data quality, expedite collection, and decrease risk of missing data in future studies. Lastly, we did not record individual-level program attendance, which limited our ability to evaluate an associated intervention dose-response effect. We plan to further investigate this question in larger cohort studies by recording individual-level attendance data for all *Chamas* participants.

### Implications for practice

Our findings demonstrated participation in a group-based health education and microfinance program during the antenatal and postpartum period was associated with higher odds of facility-based delivery compared to the standard of care. Program participation also promoted the practice of other positive MNCH behaviors. Local MOH representatives and policymakers should consider *Chamas* when seeking alternative strategies to promote positive MNCH behaviors in resource-limited settings.

Though these results highlight *Chamas’* potential, it is important to acknowledge quality and availability of services may impair translated improvement in health outcomes. The intent to deliver in a health facility, for instance, may be compromised if trained providers are in short-supply or inadequately prepared to deliver life-saving interventions. Participant-driven behaviors, such as exclusively breastfeeding, may conversely yield more consistent outcomes as these behaviors are less dependent on external services. By highlighting this distinction, we underscore the importance of not only promoting positive behaviors but also bolstering the quality of services provided to those who seek them.

Currently, the *Chamas* program is gradually transitioning leadership to the Busia County MOH, whose representatives have supported the program’s growth and sustainability. Now in its sixth year, *Chamas* has worked with over 1100 mother-infant dyads to promote positive MNCH behaviors across the county. In the near future, we hope to clarify the program’s impact on MNCH outcomes on a population-level by pursuing a cluster randomized controlled trial.

## Conclusions

In summary, *Chamas* participation during pregnancy and postpartum was associated with a five-fold increase in the odds of facility-based delivery compared to receiving the standard of care in rural western Kenya. Larger proportions of program participants also practiced other positive MNCH behaviors. This program demonstrates potential to achieve population-level MNCH benefits; however, a larger study is needed to validate this observed effect.

## Supplementary information

**Additional file 1.** Data collection forms and questionnaire guides for intervention and control cohorts.

## Data Availability

The datasets used and/or analyzed during the current study are available from the corresponding author on reasonable request.

## Abbreviations

AMPATH: Academic Model Providing Access to Healthcare
ANC: Antenatal Care
*Chamas*: *Chamas for Change* Program
CHEW: Community Health Extension Worker
CHV: Community Health Volunteer
EBF: Exclusively Breastfeed
FP: Family Planning
GA: Gestational Age
GISHE: Group Integrated Savings for Health and Empowerment
GOK: Government of Kenya
IMR: Infant Mortality Rate
MCH: Maternal and Child Health
MMR: Maternal Mortality Ratio
MNCH: Maternal, Newborn, and Child Health
MOH: Ministry of Health
NMR: Neonatal Mortality Rate
OPV0: Oral Polio Vaccine 0
SBA: Skilled Birth Attendant
SDG: Sustainable Development Goal
WHO: World Health Organization
