# Supplementary material for: Promoting positive maternal, newborn, and child health behaviors through a group-based health education and microfinance program: a prospective matched cohort study in western Kenya
Source: BMC Pregnancy Childbirth. 2020 May 12;20:288. doi: 10.1186/s12884-020-02978-w (PMC7216653; doi:10.1186/s12884-020-02978-w)
Supplement: Supplementary file 1 — Additional file 1. Data collection forms and questionnaire guides for intervention and control cohorts. [file 12884_2020_2978_MOESM1_ESM.pdf]

Individual Chama Data Collection Tool *Date Filled out:* *Name of Data Collector:*

**Identification**	**First name**	**Middle name**	**Surname**	**Village**	**Age**	
**Personal info**	**Gravidity (# pregnancies including current)**	**Parity (# living children)**	**Prior facility delivery (Y/N/NA) Before Oct. 1, 2012**	**Education level code**	**Marital status code**	**Employment code**
**Chama membership info**	**Date joined chama (DD/MM/YR)**	**Date left chama (DD/MM/YR or NA if still member)**	**RH status when joined code**	**If pregnant, #months when joined**	**EDD (DD/MM/YR) or NA**	**Miscarriage of the pregnancy (Y/N/NA)**
**MCH Booklet**	**Date of delivery (DD/MM/YR or NA)**	**Place of delivery (Home, Facility name, NA)**	**Date of 1st ANC visit (DD/MM/YR or NA)**	**Facility name of 1st ANC visit**	**# ANC visits total**	**OPVO (Y/N/NA)**
**Postnatal (verbal report)**	**CHV 48 hr home visit (Y/N/NA)**	**Age of solid/liquid food or water introduction code**	**Infant or child <5 death between October 1, 2012-October 1, 2013 (Y/N/NA)**	**If YES to infant/child death, record age of child using code**	**Maternal death within 6 weeks of delivery (Y/N)**	**Long term FP use code** / **If yes, √ If <6 months postpartum**
**GISE info and from GISE book**	**Participating in GISE (Y/N)**	**If no, did you drop out from GISE? (Y/N)**	**# loans obtained from chama**	**Use of loans code (list more than 1)**	**# of meetings attended since joining**	

**Codes**	**0**	**1**	**2**	**3**	**4**	**5**	**6**	**7**
Education level	None	Primary 1-3	Primary 4-8	Secondary 1-2	Secondary 3-4	College certificate	College diploma	University
Marital Status	Single	Married	Divorced	Widowed				
Employment	Housewife	Self-employed	Farmer/Fisher	Casual labour	Civil servant	Health worker	Student	Other
RH status when joined	NeverPregnant	Pregnant	Postnatal <6 wks	Postnatal 6w-6mo	Postnatal 6mo-1yr	Postnatal > 1yr		
Age of infant/child	Stillborn	<1 month	1 month-12 mo	1 year	2 years	3 years	4 years	5 years
Food/water intro	< 1month	1 month	2 months	3 months	4 months	5 months	6 months	≥7 months
Long term FP method	None	Implanon/Jadelle	IUCD	BTL	Vasectomy			
Use of loans	School fees	Sick child	Pregnancy	Household expenses	Start business	Expand business	Other	

Individual Chama Data Collection Tool Key for Chama Women

1. **Identification Row**
    a. **First name:** Per identification card if available
    b. **Middle name:** Per identification card if available
    c. **Surname:** Per identification card if available
    d. **Village:** Name of the village in which the woman lived in October 2012
    e. **Age (in years at time joined):**
    Chama group: Obtain the age of the woman at the time she joined the chama in 2012

2. **Personal Info**
    a. **Gravidity (if pregnant including current)**
    Ask: How many times have you been pregnant?
        i. Both groups: This includes any miscarriages, abortions, or loss of fetus during the pregnancy.
        ii. Chama group: Count all pregnancies of the woman at the time she joined the chama including the pregnancy at the time of joining.
    b. **Parity (# living children)**
    Ask: How many children did you have alive as of January 2013?
        i. Both groups: This includes only children who are alive as of January 2013.
        ii. For women who were pregnant upon joining chama, their gravidity and parity cannot be the same unless the woman gave birth to twins in the past.
        iii. Non-pregnant chama women on joining will likely have the same gravidity and parity because they were not currently pregnant.
    c. **Prior facility delivery (Y/N/NA) (Before Oct. 1, 2012)**
    Ask: Had you ever delivered in the hospital before October 2012?
        i. NA: Use this code for women who have never delivered in the past.

    d. **Education level code**
    Key as follows:

**Codes**	**0**	**1**	**2**	**3**	**4**	**5**	**6**	**7**
Education level	None	Primary 1-3	Primary 4-8	Secondary 1-2	Secondary 3-4	College certificate	College diploma	University

    e. **Marital Status Code**
    Note: Marital status as of October 1, 2012.

**Codes**	**0**	**1**	**2**	**3**	**4**	**5**	**6**	**7**
Marital Status	Single	Married	Divorced	Widowed				

    f. **Employment Code**
    Note: Employment status of the woman as of October 1, 2012.

**Codes**	**0**	**1**	**2**	**3**	**4**	**5**	**6**	**7**
Employment	Housewife	Self-employed	Farmer/Fisher	Casual labour	Civil servant	Health worker	Student	Other

3. **Chama Membership Info**
    a. **Date joined chama (DD/MM/YR)**
    Ask: When did you join chama?
    Clarification: If day is unknown, check chama records first and if still unknown, put month and year only.
    b. **Date left chama (DD/MM/YR or NA if still member)**
    Did you stay in the chama up to the share out period?
        i. If not, when did you stop attending chamas?
        ii. If the woman shared out, put the date of the share out.

c. **RH status when joined code**
Ask: Were you pregnant when you joined a chama?
   i. If yes, fill in 0.
   ii. If no, ask: did you join with a baby? How old was the baby?
   iii. Fill in appropriate code based on above questions.

**Codes**	**0**	**1**	**2**	**3**	**4**	**5**	**6**	**7**
RH status when joined	Never Pregnant	Pregnant	Postnatal <6 wks	Postnatal 6w-6mo	Postnatal 6mo-1yr	Postnatal > 1yr		

d. **If pregnant, # months when joined**
This will be left blank, and we will calculate this based on EDD and Date joined chama.

e. **EDD (DD/MM/YR) or NA**
Look: Look in the MCH booklet for an EDD recorded by the nurse.
Ask: When you were pregnant at the end of 2012, what date did the CHV or nurse say you would deliver?

f. **Miscarriage of the pregnancy (Y/N/NA)**
Ask: When you were pregnant at the end of 2012, did you carry the pregnancy to delivery? If no, what happened?
   i. If you are unsure to write yes or no, you may write a comment.

4. **MCH Booklet: Please try to obtain this information from MCH booklet, but if the booklet is unavailable, please try to obtain historic data.**
****This row should not be filled out if the woman did not join the chama pregnant.**

a. **Date of Delivery (DD/MM/YR or NA)**
Look: Check the MCH book to determine the date of delivery.
If information not available, ask: When you were pregnant at the end of 2012, what day did you deliver the baby?
   i. If she miscarried, you will write NA.
   ii. If she delivered a baby that did not live, please record the date.

b. **Place of delivery (Home, Facility name, NA)**
Look: Check the MCH book to determine the place of delivery.
If information not available, ask: With your pregnancy at the end of 2012, where did you deliver?
   i. NA- Answer for a woman who miscarried/aborted.
   ii. If she delivered in transit or neither in home or in the facility, please write the location.

c. **Date of 1st ANC visit (DD/MM/YR or NA)**
Look: Check the MCH book to determine the date of the 1st ANC visit.
If information not available, ask: With your pregnancy at the end of 2012, what day did you first go to clinic for antenatal care (ANC)?
   i. NA- She never attended ANC clinic.
   ii. If she does not know the date and it is not in the book, try to obtain the month and year.

d. **Facility name of 1st ANC visit**
Look: Check the MCH book to determine the place of the 1st ANC visit.
If information not available, ask: For this visit, which clinic did you attend?

e. **# ANC visits total**
Look: Check the book and determine the number of total ANC booklets by counting the visits.

If not available in book, ask: For this same pregnancy, how many times did you go to the clinic for pregnant mothers (ANC)?

f. **OPV 0 (Y/N/NA)**
Look: Check the book and determine if the infant received OPV 0.
If information not available, ask: Was your child given drops of medicine in the mouth for prevention of polio at birth or within two weeks of age?
    i. NA- Answer for a woman who miscarried/aborted, had a stillbirth or had a newborn die at delivery.

5. **Postnatal (verbal report)**
****This row should not be filled out if the woman did not join the chama pregnant or if the woman lost the pregnancy or baby.**

a. **CHV 48 hr home visit (Y/N/NA)**
Ask: With your delivery for your pregnancy at the end of 2012, did a CHV visit you in the home to see you and the newborn within 48 hours of life?
    i. NA- The woman does not know who a CHV is or does not know if one visited.

b. **Age of solid/liquid food or water introduction code**
Ask: At what age was your infant, when he/she first received any food, fluids or water other than breast milk?

**Codes**	**0**	**1**	**2**	**3**	**4**	**5**	**6**	**7**
Food/water intro	< 1month	1 month	2 months	3 months	4 months	5 months	6 months	≥7 months

c. **Infant or child <5 death between October 1, 2012-October 1, 2013 (Y/N/NA)**
Ask: Did a child or infant of yours under 5 years of age die between October 1, 2012 and October 1, 2013?
    i. NA- A woman has had no children.

d. **If YES to infant/child death, record age of child using code**
Ask: How old was your child when he/she died?
    i. Leave blank if there was no child death between October 1, 2012-October 1, 2013.

**Codes**	**0**	**1**	**2**	**3**	**4**	**5**	**6**	**7**
Age of infant/child	Stillborn	<1 month	1 month-12 mo	1 year	2 years	3 years	4 years	5 years

e. **Maternal death within 6 weeks of delivery (Y/N)**
If not speaking with the mother, ask: Did the mother die during her pregnancy or within 6 weeks after her delivery?

f. **Long term FP use code**
Ask: Were you using any family planning options as of October 1, 2013?
    i. If yes, what option? If it is a long-term method in the list below, please fill in the appropriate code.
    ii. If no, continue to next question.

**Codes**	**0**	**1**	**2**	**3**	**4**	**5**	**6**	**7**
Long term FP method	None	Implant Implanon/Jadelle	IUCD (Intrauterine contraceptive device)	BTL (Bilateral Tubal Ligation)	Vasectomy			

g. **If yes, √ If <6 months postpartum**
If they are on one of the coded long-term family planning methods, ask: Was your family planning method obtained within 6 months of your pregnancy?
    i. If yes, place a check mark.
    ii. If no, write N.
    iii. If not on long-term FP, write NA.

6. **GISHE Info (from GISHE book)**
    a. **Participating in GISHE (Y/N)**
    Ask: Did you participate in GISHE in your chama until the share out in 2013?

    b. **If no, did you drop out from GISHE? (Y/N)**
    c. **# loans obtained from chama**
    Look: Look in GISHE record to obtain the number of loans obtained by this specific woman.
    If GISHE record is unavailable, ask: How many loans did you obtain between October 1, 2012 and share out in 2013?
        i. Write 0 if she did not obtain any loans
        ii. Write NA if she was not enrolled in GISHE.
    d. **Use of loans code (list more than 1)**
    Ask: How did you use your loans from GISHE?

**Codes**	**0**	**1**	**2**	**3**	**4**	**5**	**6**	**7**
Use of loans	School fees	Sick child	Pregnancy	Household expenses	Start business	Expand business	Other	

    e. **# of meetings attended since joining**
    Look: Count in the GISHE record how many meetings this specific woman attended.

Control Data Collection Tool *Date Filled out:* *Name of Data Collector:*

Name of control woman written from list: *1st ANC Facility:*

**Identification**	**First name**	**Middle name**	**Surname**	**Village**	**Age**		
**Personal info**	**Gravidity (# pregnancies including current)**	**Parity (# living children)**	**Prior facility delivery (Y/N/NA) Before Oct. 1, 2012**	**Education level code**	**Marital status code**	**Employment code**	
**Pregnancy Info**	**EDD (DD/MM/YR) or NA**	**Miscarriage of the pregnancy (Y/N/NA)**	**Maternal death within 6 weeks of delivery (Y/N)**	**Infant or child <5 death between October 1, 2012-October 1, 2013 (Y/N/NA)**	**If YES to infant/child death, record age of child using code**		
**MCH Booklet**	**Date of delivery (DD/MM/YR or NA)**	**Place of delivery (Home, Facility name, NA)**	**Date of 1st ANC visit (DD/MM/YR or NA)**	**Facility name of 1st ANC visit**	**# ANC visits total**	**OPVO (Y/N/NA)**	
**Postnatal (verbal report)**	**CHV 48 hr home visit (Y/N/NA)**	**Age of solid/liquid food or water introduction code**	**Long term FP use code**	**If yes, √ If <6 months postpartum**			

**Codes**	**0**	**1**	**2**	**3**	**4**	**5**	**6**	**7**
Education level	None	Primary 1-3	Primary 4-8	Secondary 1-2	Secondary 3-4	College certificate	College diploma	University
Marital Status	Single	Married	Divorced	Widowed				
Employment	Housewife	Self-employed	Farmer/Fisher	Casual labour	Civil servant	Health worker	Student	Other
Age of infant/child	Stillborn	<1 month	1 month-12 mo	1 year	2 years	3 years	4 years	5 years
Food/water intro	< 1month	1 month	2 months	3 months	4 months	5 months	6 months	≥7 months
Long term FP method	None	Implanon/Jadelle	IUCD	BTL	Vasectomy			

Control Data Collection Tool Key for Chama Program

***Each woman interviewed must be pre-identified from the control list. Do not continue with the interview if it is not clear that this is the woman identified as a control case.**
****Every woman interviewed must first be consented to obtain the information.**
*****Fill in the information at the top based on the information present before reaching the home.**

1. **Identification Row**
    a. **First name:** Per identification card if available
    b. **Middle name:** Per identification card if available
    c. **Surname:** Per identification card if available
    d. **Village:** Name of the village in which the woman lived in October 2012
    e. **Age (in years at time joined):**
    Control group: obtain the age of the woman in October of 2012

2. **Personal Info**
    a. **Gravidity (if pregnant including current)**
    Ask: How many times have you been pregnant?
        i. Both groups: This includes any miscarriages, abortions, or loss of fetus during the pregnancy.
        ii. Control group: Count all pregnancies of the woman as of January 2013 including the pregnancy that pregnancy.
    b. **Parity** (**# living children)**
    Ask: How many children did you have alive as of January 2013?
        i. Both groups: This includes only children who are alive as of January 2013.
        ii. For control women pregnant between October and December 2012, their gravidity and parity cannot be the same unless the woman gave birth to twins in the past.
    c. **Prior facility delivery (Y/N/NA)** (Before Oct. 1, 2012)
    Ask: Had you ever delivered in the hospital before October 2012?
        i. NA: Use this code for women who have never delivered in the past.
    d. **Education level code**
    Key as follows:

**Codes**	**0**	**1**	**2**	**3**	**4**	**5**	**6**	**7**
Education level	None	Primary 1-3	Primary 4-8	Secondary 1-2	Secondary 3-4	College certificate	College diploma	University

    e. **Marital Status Code**
    Note: Marital status as of October 1, 2012.

**Codes**	**0**	**1**	**2**	**3**	**4**	**5**	**6**	**7**
Marital Status	Single	Married	Divorced	Widowed				

    f. **Employment Code**
    Note: Employment status of the woman as of October 1, 2012.

**Codes**	**0**	**1**	**2**	**3**	**4**	**5**	**6**	**7**
Employment	Housewife	Self-employed	Farmer/Fisher	Casual labour	Civil servant	Health worker	Student	Other

3. **Pregnancy Information**
    a. **EDD (DD/MM/YR) or NA**
    Look: Look in the MCH booklet for an EDD recorded by the nurse.
    Ask: When you were pregnant at the end of 2012, what date did the CHV or nurse say you would deliver?

b. **Miscarriage of the pregnancy (Y/N/NA)**
Ask: When you were pregnant at the end of 2012, did you carry the pregnancy to delivery? If no, what happened?
   i. If you are unsure to write yes or no, you may write a comment.

c. **Maternal death within 6 weeks of delivery (Y/N)**
If not speaking with the mother, ask: Did the mother die during her pregnancy or within 6 weeks after her delivery?

d. **Infant or child <5 death between October 1, 2012-October 1, 2013 (Y/N/NA)**
Ask: Did a child or infant of yours under 5 years of age die between October 1, 2012 and October 1, 2013?
   i. NA- A woman has had no children.

e. **If YES to infant/child death, record age of child using code**
Ask: How old was your child when he/she died?
   i. Leave blank if there was no child death between October 1, 2012-October 1, 2013.

**Codes**	**0**	**1**	**2**	**3**	**4**	**5**	**6**	**7**
Age of infant/child	Stillborn	<1 month	1 month-12 mo	1 year	2 years	3 years	4 years	5 years

4. **MCH Booklet: Please try to obtain this information from MCH booklet, but if the booklet is unavailable, please try to obtain historic data.**

a. **Date of Delivery (DD/MM/YR or NA)**
Look: Check the MCH book to determine the date of delivery.
If information not available, ask: When you were pregnant at the end of 2012, what day did you deliver the baby?
   i. If she miscarried, you will write NA.
   ii. If she delivered a baby that did not live, please record the date.

b. **Place of delivery (Home, Facility name, NA)**
Look: Check the MCH book to determine the place of delivery.
If information not available, ask: With your pregnancy at the end of 2012, where did you deliver?
   i. NA- Answer for a woman who miscarried/aborted.
   ii. If she delivered in transit or neither in home or in the facility, please write the location.

c. **Date of 1st ANC visit (DD/MM/YR or NA)**
Look: Check the MCH book to determine the date of the 1st ANC visit.
If information not available, ask: With your pregnancy at the end of 2012, what day did you first go to clinic for antenatal care (ANC)?
   i. NA- She never attended ANC clinic.
   ii. If she does not know the date and it is not in the book, try to obtain the month and year.

d. **Facility name of 1st ANC visit**
Look: Check the MCH book to determine the place of the 1st ANC visit.
If information not available, ask: For this visit, which clinic did you attend?

e. **# ANC visits total**
Look: Check the book and determine the number of total ANC booklets by counting the visits.

If not available in book, ask: For this same pregnancy, how many times did you go to the clinic for pregnant mothers (ANC)?

f. **OPV 0 (Y/N/NA)**
Look: Check the book and determine if the infant received OPV 0.
If information not available, ask: Was your child given drops of medicine in the mouth for prevention of polio at birth or within two weeks of age?
   i. NA- Answer for a woman who miscarried/aborted, had a stillbirth or had a newborn die at delivery.

5. **Postnatal (verbal report)**
****This row should not be filled out if the woman lost her pregnancy before the third trimester.**

a. **CHV 48 hr home visit (Y/N/NA)**
Ask: With your delivery for your pregnancy at the end of 2012, did a CHV visit you in the home to see you and the newborn within 48 hours of life?
   i. NA- The woman does not know who a CHV is or does not know if one visited.

b. **Age of solid/liquid food or water introduction code**
Ask: At what age was your infant, when he/she first received any food, fluids or water other than breast milk?

**Codes**	**0**	**1**	**2**	**3**	**4**	**5**	**6**	**7**
Food/water intro	< 1month	1 month	2 months	3 months	4 months	5 months	6 months	≥7 months

c. **Long term FP use code**
Ask: Were you using any family planning options as of October 1, 2013?
   i. If yes, what option?
   ii. If no, continue to next question.

**Codes**	**0**	**1**	**2**	**3**	**4**	**5**	**6**	**7**
Long term FP method	None	Implant Implanon/Jadelle	IUCD (Intrauterine contraceptive device)	BTL (Bilateral Tubal Ligation)	Vasectomy			

d. **If yes, √ If <6 months postpartum**
If they are on one of the coded long-term family planning methods, ask: Was your family planning method obtained within 6 months of your pregnancy?
   i. If yes, place a check mark.
   ii. If no, write N.
   iii. If not on long-term FP, write NA.
